# Relationship Between Diet Quality and Statin Use Among Adults With Metabolic Syndrome From the CARTaGENE Cohort

**DOI:** 10.1016/j.cjco.2023.09.014

**Published:** 2023-09-29

**Authors:** Amélie Bélanger, Clémence Desjardins, Lise Leblay, Mathieu Filiatrault, Olivier Barbier, Anne Gangloff, Jacinthe Leclerc, Jean Lefebvre, Arsène Zongo, Jean-Philippe Drouin-Chartier

**Affiliations:** a NUTRISS (Nutrition, Health and Society) Research Centre, Institute on Nutrition and Functional Foods, Laval University, Québec City, Québec, Canada; b Faculty of Pharmacy, Laval University, Québec City, Québec, Canada; c CHU de Québec-Laval University Research Center, Québec City, Québec, Canada; d Faculty of Medicine, Laval University, Québec City, Québec, Canada; e Research Center, Institut Universitaire de Cardiologie et de Pneumologie de Québec-Université Laval, Québec City, Québec, Canada

## Abstract

**Background:**

In metabolic syndrome (MetS), cardiovascular disease (CVD) risk reduction relies on the complementary use of diet and lipid-lowering medication. Evidence suggests that initiating such medication may impede diet quality. The objective of this study was to evaluate the relationship between diet quality and statin use among adults with MetS and free of CVD from the Province of Québec.

**Methods:**

This cross-sectional study included 2481 adults with MetS (40-69 years of age) from the CARTaGENE Québec population-based cohort, of whom 463 self-reported using statin monotherapy. Diet was assessed using the Canadian Dietary History Questionnaire II, a food- frequency questionnaire, and diet quality was assessed using the Alternative Healthy Eating Index (AHEI).

**Results:**

In multivariable-adjusted linear regression models, statin users had lower AHEI (%) compared with nonusers (users: 40.0; 95% confidence interval [CI], 38.9, 41.2 vs nonusers: 41.2; 95% CI, 40.4, 42.0; *P* = 0.03] because of a lower consumption of vegetables and whole grains. Stratified interaction analyses showed that the lower diet quality among statin users was mostly prevalent among men aged ≥ 50 years and women aged ≥ 60 years, among individuals with annual household incomes of < $50,000 and persons who self-reported history of high blood pressure.

**Conclusions:**

In this cohort of adults with MetS from Quebéc, the use of statin monotherapy in primary prevention of CVD was associated with a slightly lower diet quality. These data suggest suboptimal complementarity between diet quality and use of cholesterol-lowering medication in primary prevention of CVD in MetS.

## Résumé

**Contexte:**

Dans le syndrome métabolique, la réduction du risque de maladie cardiovasculaire repose sur la complémentarité entre une saine alimentation et l'utilisation d’hypolipidémiants. Des évidences suggèrent que l'initiation d’un traitement médicamenteux hypolipémiant influencerait négativement la qualité de l'alimentation. Cette étude avait pour objectif de déterminer la relation entre la qualité de l’alimentation et l’utilisation de statines chez des adultes avec un syndrome métabolique, mais sans maladie cardiovasculaire, au Québec.

**Méthodologie:**

Cette étude transversale comptait 2481 adultes avec un syndrome métabolique (âgés de 40 à 69 ans) provenant de la cohorte CARTaGENE, représentative de la population du Québec, dont 463 sujets ayant autodéclaré qu’ils prenaient une statine en monothérapie. L’alimentation des sujets a été évaluée à l’aide du *Canadian Dietary History Questionnaire II,* un questionnaire de fréquence alimentaire, et la qualité de l’alimentation a été évaluée à l’aide de l’*Alternative Healthy Eating Index* (AHEI).

**Résultats:**

Dans des modèles de régression linéaire multivariée, les utilisateurs de statines ont présenté un indice AHEI (%) plus faible en comparaison aux non-utilisateurs (utilisateurs : 40,0; intervalle de confiance [IC] à 95 % : 38,9-41,2 *vs* non-utilisateurs : 41,2; IC à 95 % : 40,4-42,0; *p* = 0,03), en raison d’une plus faible consommation de légumes et de grains entiers. Selon des analyses d’interaction stratifiées, la plus faible qualité nutritionnelle chez les utilisateurs de statines était particulièrement prévalente chez les hommes de ≥ 50 ans et les femmes de ≥ 60 ans, chez les personnes dont le revenu annuel du ménage était < 50 000 dollars et chez les personnes ayant autodéclaré des antécédents d’hypertension.

**Conclusions:**

Dans cette cohorte d’adultes du Québec avec un syndrome métabolique, l’utilisation de statines en monothérapie dans la prévention des maladies cardiovasculaires était associée à une alimentation dont la qualité était légèrement plus faible. Ces données suggèrent une complémentarité sous-optimale entre la qualité de l’alimentation et l’utilisation d’hypocholestérolémiants en prévention des maladies cardiovasculaires chez les personnes avec un syndrome métabolique**.**

Cardiovascular diseases (CVDs) are the leading causes of death and substantially contribute to loss of health and excess health system costs worldwide.^1^ Canada is no exception, as approximately 14 people die from CVD every hour.^2^ In that regard, > 20% of Canadian adults are affected by metabolic syndrome (MetS), a cluster of metabolic anomalies that includes abdominal obesity, high blood pressure, insulin resistance and dyslipidemia, and significantly increases CVD risk.^3^^,^^4^ In MetS, primary prevention of CVD relies on the normalization of risk factors.^4^ As such, the use of statin, a cholesterol-lowering drug, in conjunct with a heart-healthy diet is the recommended first line therapy by the Canadian Cardiovascular Society (CCS).^5^

In recent years, accumulating evidence showing that statin initiation is associated with unfavourable lifestyle changes has been reported.^6^ For instance, in a prospective cohort study from Finland, individuals who initiated statins over the first decade of the 2000s subsequently experienced greater declines in physical activity practice compared with noninitiators.^7^ Similarly, over the same period of time, data from the National Health and Nutrition Examination Survey (NHANES) in the United States showed that energy and fat intake consistently increased among people using statins, whereas they remained stable among nonusers.^8^ It has been suggested that the perceived effectiveness of statins, albeit being essential to adherence to pharmacotherapy, may represent a barrier to lifestyle modification and even motivate unfavourable lifestyle changes.^9^^,^^10^ This issue warrants consideration, as nearly 50% of individuals initiating statins do not achieve the targeted reduction in plasma cholesterol and thus remain exposed to an important residual risk of CVD.^11^ Moreover, failure to achieve such target may justify medication intensification, which increases the risks of both adverse effects and nonadherence, further fueling this cycle. Still, to our knowledge, no study has ever assessed the agreement between diet quality and statin use in primary prevention of CVD in Canada.

The objective of this study was to assess the relationship between diet and statin use among adults with MetS and free of CVD from the Province of Québec. Specifically, we evaluated the relationship among diet quality, dietary intakes, and statin use and explored individual characteristics underlying this relationship. We also assessed the relationship among diet quality, statin use, and plasma lipids. This work was restricted to a cohort from Québec because of provincial specificity of medication insurance programs and primary care organization.^12^^,^^13^

## Methods

The protocol was reviewed and approved by Laval University Ethics Committee as well as CARTaGENE Sample and Data Access Committee.

### Study design and population

This study is a cross-sectional analysis conducted within the CARTaGENE Québec population-based cohort (Canada).^14^ A total of 43,038 Québec residents, aged between 40 and 69 years, were recruited to participate in CARTaGENE during 2 phases (A: n = 19,069, 2009-2010; B: n = 23,969, 2013-2014). Participants were randomly solicited from provincial health insurance registries to represent the Québec population according to 2006 census data based on age, sex, and area of residence for population density. CARTaGENE adhered to the Declaration of Helsinki, and all participants signed informed consent forms.

The current study leveraged data from Phase A, as comprehensive diet assessment was only conducted among participants of this phase. Phase A participants completed an in-person interviewer-administered health questionnaire, which included items on lifestyle, history of diseases, medication, and socioeconomic information (2009-2010).^15^^,^^16^ Plasma samples and physical measures were also collected during the interview. In 2012, participants from Phase A were invited to complete a food-frequency questionnaire (FFQ) from home, which ended up being completed and returned by ∼10,000 people.

For the current analysis, inclusion criteria were (1) having MetS (per the harmonized definition)^4^; (2) having adequately completed the FFQ (ie, < 40% of blank items); (3) having reported plausible energy intakes (ie, women: 500-3500 kcal per day; men: 800-4200 kcal per day); and (4) having provided a blood sample from which plasma lipids were measured. Individuals with personal history of CVD, cancer, or diabetes or using nonstatin lipid-lowering medication (eg, ezetimibe) were excluded. A total of 2481 participants were included, of whom 463 were using statins as a lipid-lowering monotherapy (Supplemental Fig. S1).

### Assessment of diet and diet quality

Diet was assessed using the Canadian Dietary History Questionnaire II (C-DHQ II).^17^, ^18^, ^19^, ^20^ This FFQ was initially developed and validated by the US National Cancer Institute.^17^, ^18^, ^19^, ^20^ The US version has been modified to reflect food availability, brand names, nutrition composition, and food fortification in Canada according to the 24-hour dietary recall data collected in the national 2004 Canadian Community Health Survey.^20^ The C-DHQ II assesses the frequency of consumption of 153 foods and the portion size usually consumed in the 12 months preceding its completion.

Diet quality was graded using the Alternative Healthy Eating Index (AHEI) score.^21^ The AHEI was created based on intakes of foods and nutrients that have been consistently associated with lower CVD risk.^21^ The score is calculated from intakes of 10 dietary components. Intakes of vegetables, fruits, whole grains, nuts and legumes, fatty fish, and polyunsaturated fatty acids are positively scored. Intakes of red and processed meat, sugar-sweetened beverages and fruit juices, trans fat, and sodium are negatively scored. Each component is scored individually from 0 (worst) to 10 (best) based on a priori defined thresholds, except for sodium, for which scoring relies on the sample distribution.^21^ The AHEI ranges from 0 to 100 (maximum adherence).

### Assessment of statin use

Information on statin use was obtained from the health questionnaire in which participants had to self-report the types and dosages of the medication they used. A previous analysis conducted within the CARTaGENE cohort demonstrated the high agreement (κ > 0.80) between self-reported information on medication acting on the cardiovascular system—such as statins—and drug-claims data.^22^

### Assessment of plasma lipids

Concentrations of total cholesterol (Total-C), high-density lipoprotein cholesterol (HDL-C) and triglycerides (TGs) were measured from the fasting plasma sample collected during the in-person interview. Low-density lipoprotein cholesterol (LDL-C) concentrations were calculated using the Friedewald formula.^23^ Other circulating biomarkers of cardiometabolic health (eg, plasma glucose) were also measured from this sample. Upon collection, samples were sent to clinical diagnostic laboratories for immediate hematologic and biochemical analysis. Quality assurance tests in the optimization phase demonstrated that all biochemical parameters were measured with test-retest reliability > 90%.^14^

### Assessment of covariables

The International Physical Activity Questionnaire was used to assess physical activity.^24^ Information on tobacco smoking was self-reported. Three sequential readings of systolic and diastolic blood pressure were obtained by oscillometry with an automated device (Press-Mate Prodigy II Vital Signs Monitor OM-2200, Omron, Kyoto, Japan). Waist circumference was measured twice (Seca 200 measuring tape, Seca GmbH, Hamburg, Germany). Participants’ height was also measured twice (Seca 214 portable stadiometer). A digital scale was used to measure the weight of each participant. Body mass index (BMI) was calculated from weight and height. The Framingham Risk Score (FRS) was calculated using the CCS algorithm.^25^

### Statistical analyses

Statistical analyses were performed using SAS software version 9.4 (SAS Institute, Cary, NC). All statistical tests were 2-sided with a significance threshold set at *P* < 0.05. Before analyses, we imputed missing values of covariables, using the median for continuous variables or the most prevalent value for categorical variables (Supplemental Table S1).

We first compared diet quality and dietary intakes according to statin use, using linear regression models (GLM procedure). Diet quality was assessed using the AHEI (in percent). For dietary intakes, we modelled both the AHEI subscore (per 10 points) as well as daily intakes (amount per day) of each AHEI component, per statin use. Models were adjusted for gender (women/men), age (years), annual household income (< $10,000; $10,000-$24,999; $25,000-$49,999; $50,000-$74,999$; $75,000-$99,999; $100,000-$149,999$; $150,000-$199,999; > $200,000), smoking status (never/past/current), physical activity level (low/moderate/high), self-reported history of high blood pressure (no/yes), BMI (kg/m^2^), energy intake (kcal per day), and alcohol consumption (grams per day). In addition, we compared diet quality between users and nonusers but by separating nonusers according to whether they were meeting CCS criteria for statin initiation or not (nonusers not meeting statin criteria vs nonusers meting statin criteria vs statin users).^5^ In these analyses, Tukey-Kramer’s multiple comparison test was used to identify between-group significant statistical differences. Finally, we explored whether the potential difference in the AHEI associated with statin use (no vs yes) differed according to prestipulated characteristics: gender (women vs men), age (men < 50 and women < 60 years vs men ≥ 50 and women ≥ 60 years), education level (high school or less vs college or university), annual household income (< $50,000 vs ≥ $50,000), smoking status (never vs past vs current), BMI (< 30 kg/m^2^ vs ≥ 30 kg/m^2^), self-reported history of high blood pressure (no vs yes), self-reported history of high blood cholesterol (no vs yes), or FRS (low vs moderate vs high). The age stratification sex/gender-specific rationale relies on the thresholds associated with higher age-related CVD risk.^26^^,^^27^ Evidence of interactions was assessed using the *P* value of the cross-product term between statin use and the stratification variable.

Second, we assessed the relationship between statin use (no/yes), the AHEI (in percent), and plasma lipids (ie, Total-C, LDL-C, HDL-C, non-HDL-C, TGs), also using linear regression models. The same covariable structure as described here was used. Analyses with statin use as the main independent variable were adjusted for the AHEI and vice versa. We also tested whether the relationship between the AHEI and plasma lipids differed between statin users and nonusers using interaction tests. Finally, we used logistic regression models (LOGISTIC procedure) to calculate the odds ratios of having plasma concentrations of non-HDL-C or LDL-C below target levels established by the CCS for primary prevention of CVD (ie, non-HDL-C < 2.60 mmol/L; LDL-C < 2.00 mmol/L)^5^ associated with statin use and the AHEI, respectively. In this analysis, statin use and the AHEI were included simultaneously in the model as independent variables, along with the covariables listed here. For all linear regression models, the normality of the models was assessed using the distribution of the scaled residual values. Whenever these were not normally distributed, we used the Box-Cox approach (TRANSREG procedure) to identify the type of transformation that would allow normalization of the scaled residual values of the models.

## Results

Table 1 presents characteristics of the 2481 participants with MetS included in the study according to statin use. Participants using statin (n = 463) were older and more likely to be men and past smokers compared with nonusers. MetS diagnostic criteria were equally distributed among statin users and nonusers, with the exception that those using statins were more likely to have high blood pressure or to use antihypertensive medication. Individuals using statins were more likely to have a moderate or high risk of CVD per the FRS. Among statin users, atorvastatin and rosuvastatin were the most used statins. In the 2 groups, the AHEI was < 45%.

**Table 1:** Characteristics of the 2481 participants with metabolic syndrome included in the study according to statin use

Characteristics	Participants not using statins (n = 2018)	Participants using statins (n = 463)
Age, years	55.1 ± 7.6	59.5 ± 6.8
Sex/gender, n (%)		
Female	1093 (54.2)	200 (43.2)
Male	925 (45.8)	263 (56.8)
Education level, n (%)		
High school or less	490 (24.3)	130 (28.1)
College	684 (33.9)	160 (34.6)
University	844 (41.8)	173 (37.4)
Annual household income, n (%)		
< $50,000	680 (33.7)	140 (30.2)
$50,000-$99,999	866 (42.9)	222 (48.0)
≥ $100,000	472 (23.4)	101 (21.8)
MetS criteria, n (%)		
Abdominal obesity (men: ≥ 94 cm; women: ≥ 80 cm)	1805 (89.4)	424 (91.6)
High blood pressure (≥ 130/85 mm Hg) or medication use	1312 (65.0)	375 (81.0)
High triglycerides (≥ 1.7 mmol/L)	1511 (74.9)	344 (74.3)
Low HDL-C (men: < 1.0 mmol/L; women: < 1.3 mmol/L)	1459 (72.3)	307 (66.3)
High blood glucose (≥ 5.6 mmol/L)	1104 (54.7)	239 (51.6)
Smoking status, n (%)		
Never	859 (42.6)	149 (32.2)
Past	844 (41.8)	237 (51.2)
Current	315 (15.6)	77 (16.6)
Physical activity, n (%)		
Low	343 (17.0)	89 (19.2)
Moderate	895 (44.4)	190 (41.0)
High	780 (38.7)	184 (39.7)
Waist circumference, cm	98.6 ± 12.3	101.2 ± 11.1
Body mass index, kg/m^2^	29.4 ± 5.3	29.7 ± 4.5
Statin type use, n (%)		
Atorvastatin	NA	258 (55.7)
Lovastatin	NA	2 (0.4)
Pravastatin	NA	28 (6.0)
Rosuvastatin	NA	148 (32.0)
Simvastatin	NA	27 (5.8)
Framingham risk score, n (%)		
Low (< 10%)	893 (44.3)	158 (34.1)
Moderate (10%-19%)	700 (34.7)	176 (38.0)
High (≥ 20%)	425 (21.1)	129 (27.9)
Alternate healthy eating index, %	41.7 ± 10.2	40.9 ± 10.2
Plasma lipids		
Total cholesterol, mmol/L	5.43 ± 0.97	4.45 ± 0.82
LDL-cholesterol, mmol/L	3.32 ± 0.84	2.36 ± 0.69
HDL-cholesterol, mmol/L	1.08 ± 0.30	1.05 ± 0.29
Non-HDL-cholesterol, mmol/L	4.35 ± 0.92	3.40 ± 0.74
Triglycerides, mmol/L	2.23 ± 0.91	2.27 ± 0.95

Continuous variables are presented as means ± standard deviation (SD). Categorical variables are presented as count (percent).

HDL, high-density lipoprotein; MetS, metabolic syndrome; NA, not applicable.

Differences in diet quality and dietary intakes between participants not using a statin and those using this medication are presented in Table 2. The AHEI was slightly—but significantly— lower among statin users compared with nonusers. Both the AHEI subscore associated with vegetable consumption and the daily consumption (in servings per day) of this food group were significantly lower among statin users. Statistical trends also suggested that the consumption of whole grains was lower among statin users. When nonusers were separated according to whether they met CCS criteria for statin initiation (n = 1517) or not (n = 501), similar results were observed: Statin users had slightly lower AHEI scores compared with the 2 nonuser groups that had similar AHEI scores (Supplemental Table S2). Finally, we found evidence of significant interactions underlying the difference in the AHEI between individuals using statins and those not using this medication when we stratified analyses according to participants’ age and annual household income (Table 3). The difference in diet associated with statin use was observed among men aged ≥ 50 years and women aged ≥ 60 years but not among younger individuals. Also, the difference in diet quality associated with statin use was observed among individuals with annual household incomes < $50,000 but not those with higher incomes. Statistical trends also suggested such differential associations according to gender and self-reported history of high blood pressure. Men using statins had lower diet quality than men not using statins, but this was not the case among women. Likewise, individuals who reported having high blood pressure and using statins had lower diet quality compared with those not using statins, but this difference was not observed among those who did not report having high blood pressure.

**Table 2:** Differences in diet quality and intakes among the 2481 participants with metabolic syndrome included in the study according to statin use

Dietary components	Participants not using statins (n = 2018)	Participants using statins (n = 463)	*P* value
AHEI total score (%)	41.2 (40.4, 42.0)	40.0 (38.9, 41.2)	0.03
Vegetables			
AHEI subscore (/10)	7.16 (6.97, 7.36)	6.81 (6.53, 7.08)	0.005
Servings per day	4.72 (4.47, 4.97)	4.28 (3.93, 4.63)	0.005
Fruits			
AHEI subscore (/10)	3.71 (3.49, 3.92)	3.50 (3.20, 3.80)	0.36
Servings per day	1.57 (1.46, 1.68)	1.45 (1.30, 1.60)	0.20
Whole grains			
AHEI subscore (/10)	1.40 (1.27, 1.52)	1.33 (1.16, 1.51)	0.07
Servings per day	0.77 (0.70, 0.84)	0.73 (0.63, 0.83)	0.06
Sugar and sweetened beverages			
AHEI subscore (/10)	3.28 (2.97, 3.58)	3.04 (2.61, 3.47)	0.50
Servings per day	1.85 (1.62, 2.07)	1.98 (1.67, 2.29)	0.65
Nuts and legumes			
AHEI subscore (/10)	4.35 (4.09, 4.60)	4.20 (3.84, 4.55)	0.42
Servings per day	0.57 (0.52, 0.63)	0.50 (0.42, 0.58)	0.73
Red or processed meat			
AHEI subscore (/10)	3.97 (3.78, 4.17)	3.85 (3.57, 4.12)	0.34
Servings per day	1.03 (0.99, 1.08)	1.07 (1.00, 1.14)	0.18
Trans fat			
AHEI subscore (/10)	3.34 (3.22, 3.46)	3.35 (3.18, 3.52)	0.90
Grams per day	6.14 (6.04, 6.25)	6.10 (5.94, 6.25)	0.78
Fatty fish			
AHEI subscore (/10)	3.79 (3.50, 4.09)	3.94 (3.53, 4.35)	0.45
Servings per day	0.09 (0.08, 0.11)	0.09 (0.07, 0.11)	0.88
Polyunsaturated fatty acids			
AHEI subscore (/10)	5.07 (4.92, 5.23)	5.10 (4.88, 5.32)	0.72
Grams per day	13.2 (12.9, 13.5)	13.3 (12.8, 13.7)	0.68
Sodium intake			
AHEI subscore (/10)	5.10 (4.96, 5.24)	4.94 (4.74, 5.13)	0.10
mg per day	2786 (2739, 2834)	2812 (2745, 2879)	0.20

Data are presented as adjusted mean (95% confidence interval). Multiple linear regression models were used (general linear model [GLM] procedure) with adjustments for gender, age, annual household income, smoking status, physical activity level, self-reported history of high blood pressure, body mass index, energy intake, and alcohol consumption.

AHEI, Alternative Healthy Eating Index.

**Table 3:** Alternative Healthy Eating Index (AHEI) according to statin use among the 2481 participants with metabolic syndrome included in the study stratified by key characteristics

Characteristics	Participants not using statins (n = 2018)	Participants using statins (n = 463)	*P* value (between-group)	*P* value (interaction)
Gender				
Women	41.4 (40.5, 42.3)	41.2 (39.6, 42.7)	0.99	0.13
Men	40.9 (40.0, 41.9)	39.1 (37.7, 40.5)	0.05	
Age				
Male: < 50 y; female: < 60 y	40.1 (39.2, 41.1)	41.2 (39.2, 43.1)	0.77	0.04
Male: ≥ 50 y; female: ≥ 60 y	41.8 (40.9, 42.7)	40.3 (39.0, 41.6)	0.09	
Education level				
High school or less	39.4 (38.2, 40.5)	37.8 (36.0, 39.7)	0.40	0.57
College or university	41.8 (41.0, 42.7)	40.9 (39.7, 42.2)	0.48	
Annual household income				
< $50,000	41.1 (40.2, 42.0)	38.4 (36.6, 40.1)	0.02	0.05
≥ $50,000	40.9 (40.1, 41.7)	40.5 (39.3, 41.7)	0.89	
Smoking status				
Never	42.5 (41.7, 43.4)	40.3 (38.6, 42.0)	0.12	0.18
Past	42.0 (41.2, 42.9)	41.0 (39.6, 42.4)	0.71	
Current	38.8 (37.5, 40.0)	39.2 (37.0, 41.5)	0.99	
Body mass index				
< 30 kg/m^2^	41.8 (40.9, 42.6)	40.4 (39.1, 41.8)	0.20	0.63
≥ 30 kg/m^2^	40.2 (39.2, 41.2)	39.4 (37.8, 41.0)	0.77	
Self-reported history of high blood pressure				
No	41.2 (40.3, 42.0)	40.9 (39.5, 42.3)	0.96	0.09
Yes	41.3 (40.2, 42.3)	39.2 (37.7, 40.7)	0.04	
Self-reported history of high blood cholesterol				
No	40.8 (40.0, 41.6)	39.0 (36.3, 41.6)	0.50	0.48
Yes	43.2 (41.9, 44.5)	40.2 (39.0, 41.1)	0.004	
Framingham risk score				
Low (< 10%)	40.8 (39.5, 42.0)	41.0 (39.3, 42.8)	0.99	0.12
Moderate (10-19%)	41.3 (40.3, 42.3)	40.0 (38.4, 41.6)	0.62	
High (≥ 20%)	41.5 (40.3, 42.7)	39.0 (37.0, 40.9)	0.13	

Data are presented as adjusted mean (95% confidence interval). Multiple linear regression models were used (general linear model [GLM] procedure) with adjustments for sex/gender, age, annual household income, smoking status, physical activity level, self-reported history of high blood pressure, body mass index, energy intake, and alcohol consumption. The interaction term between each stratification factor and statin use was included sequentially in the models.

Differences in plasma lipids according to statin use are presented in Table 4. Participants using a statin had lower concentrations of Total-C, LDL-C, HDL-C and non-HDL-C compared with nonusers. Regarding the relationship between diet quality and plasma lipids (Table 5), we observed a positive association between the AHEI and LDL-C concentrations, and an inverse one between the AHEI and TG levels. No evidence of relationships between the AHEI and Total-C, non–HDL-C and HDL-C was observed. The relationship between the AHEI and concentrations of non–HDL-C and TGs significantly differed between statin users and nonusers (Supplemental Table S3). A positive association between the AHEI and non-HDL-C was observed among nonusers only, whereas the negative association with TGs was only significant among statin users. Finally, statin use—but not the AHEI—was significantly associated with odds of having concentrations of LDL-C < 2.00 mmol/L (n = 205 of 2481) or non-HDL-C < 2.60 mmol/L (n = 96 of 2481) (Supplemental Table S4).

**Table 4:** Differences in plasma lipids among the 2481 participants with MetS included in the study according to statin use

Plasma lipids	Participants not using statins (n = 2018)	Participants using statins (n = 463)	*P* value
Total cholesterol (mmol/L)	5.38 (5.30, 5.45)	4.38 (4.28, 4.49)	< 0.0001
LDL-cholesterol (mmol/L)	3.29 (3.22, 3.35)	2.31 (2.21, 2.40)	< 0.0001
HDL-cholesterol (mmol/L)	1.07 (1.05, 1.09)	1.03 (1.00, 1.06)	0.03
Non–HDL-cholesterol (mmol/L)	4.31 (4.23, 4.38)	3.35 (3.25, 3.45)	< 0.0001
Triglycerides (mmol/L)	2.22 (2.15, 2.29)	2.27 (2.17, 2.37)	0.40

Data are presented as adjusted mean (95% confidence interval). Linear regression models were used (general linear model [GLM] procedure) with adjustments for sex, age, annual household income, smoking status, physical activity level, self-reported history of high blood pressure, body mass index, energy intake, alternative healthy eating index, and alcohol consumption.

HDL, high-density lipoprotein; LDL, low-density lipoprotein; MetS, metabolic syndrome.

**Table 5:** Relationship between the Alternative Healthy Eating Index (AHEI) and concentrations of plasma lipids among the 2481 participants with metabolic syndrome included in the study

Plasma lipids	β (95% CI), in mmol/L, for 10-point increment in the AHEI	*P* value
Total cholesterol	0.03 (–0.01, 0.06)	0.16
LDL-cholesterol	0.04 (0.01, 0.07)	0.01
HDL-cholesterol	0.00 (–0.01, 0.01)	0.24
Non–HDL-cholesterol	0.02 (–0.01, 0.06)	0.21
Triglycerides	–0.04 (–0.08, 0.00)	0.03

Data are presented as adjusted β coefficient (95% confidence interval) associated with a 10-point increment in the AHEI. Linear regression models were used (general linear model [GLM] procedure) with adjustments for sex, age, annual household income, smoking status, physical activity level, self-reported history of high blood pressure, body mass index, energy intake, alcohol consumption, and statin use.

CI, confidence interval; HDL, high-density lipoprotein; LDL, low-density lipoprotein.

## Discussion

In this cross-sectional study in the CARTaGENE Québec population-based cohort, we observed that adults with MetS using statins had slightly lower diet quality compared with those not using such medications. This difference was because of a lower consumption of vegetables and whole grains. Subgroup analyses revealed that the lower diet quality among statin users was related to gender-, age-, socioeconomic- and health-associated factors. Overall, the difference in diet quality associated with statin use—even though it was limited in range—suggests suboptimal complementarity between diet quality and cholesterol-lowering medication use in primary prevention of CVD among adults from Québec living with MetS. This work highlights the need for improved multidisciplinary frameworks to optimize in prevention of CVD among at-risk populations.

In Canada and Québec, since the early 2000s, diet quality among adults has remained both stable and suboptimal.^28^^,^^29^ Besides, during the same period, prevalence of statin use has steadily increased, and these drugs are now among the 5 most prescribed among adults.^30^ In the Province of Québec specifically, 1 in 3 persons covered by the Public Prescription Drug Insurance uses statins.^31^ When paralleled with statistics showing that CVDs remain a leading cause of mortality and morbidity in Canada and Quebéc,^32^ these trends in diet quality and statin use suggest a lack of complementarity between nutritional and pharmacologic prevention of CVDs. However, this issue had never been thoroughly characterized in people with MetS before our study. Albeit the mean difference in AHEI between statin users and nonusers was limited in range (ie, ∼1% to 2%, depending on the subgroups), the fact that statin users had lower diet quality is problematic. Indeed, according to CVD-prevention guidelines, professional support to overcome individual, social, and systemic barriers to a healthy diet and improve diet quality should be implemented before or in conjunction with initiation of statins.^5^ We could not assess whether statin users received dietary counselling, as this information was not collected in the CARTaGENE database. However, as diet quality was lower in this group, it suggests that such support was either not implemented or ineffective to improve diet significantly. Furthermore, whether the difference in diet quality affects long-term risk of CVD could not be assessed in our study. It thus remains uncertain whether this difference is clinically meaningful or not. Still, previous analyses conducted in large prospective cohort studies reported a linear relationship between the AHEI and CVD risk, in which a 1% increase in the AHEI was associated with an approximate 1% decrease in CVD risk.^33^ Overall, our observations highlight the need for improved access to primary care and health professionals in Québec, especially given that, at the cohort level, the average AHEI was < 45% independent of statin use, which reflects a highly suboptimal diet quality; 75% of individuals not using statins were meeting conditions to initiate such therapy; and less than 10% of our sample met LDL-C or non-HDL-C targets.^5^

Our results suggest that statin use impedes diet quality, in line with conclusions of similar studies conducted in the United States^8^ and Europe.^7^ Also, this effect would be modulated by health-, age-, gender-, and socioeconomic-associated factors. This hypothesis is, in part, supported by the fact that nonusers—independent of the fact that they met CCS criteria for statin initiation—had slightly superior diet quality compared with nonusers. Indeed, using medication has been reported to be perceived not only as easier to implement but also as more effective in preventing CVDs than dietary and lifestyle modification.^9^^,^^10^^,^^34^ In addition, the fact that statin use was associated with lower diet quality among individuals who self-reported history of high blood pressure potentially positions not only statins but overall preventive medication as a potential barrier to healthy eating in primary prevention of CVD. In that regard, polypharmacy burden is known to be associated with poor medication adherence.^35^ Thus, one could suggest that polypharmacy is also associated with poor adherence to other behavioural preventive approaches such as healthy diet principles, but dedicated studies remain needed. The differential association between diet quality and statin use related to self-reported history of high blood pressure may also reflect how multimorbidity burden impedes diet quality,^36^^,^^37^ further highlighting the need for improved access to multidisciplinary frameworks in primary care. With regard to the age-related interaction, the fact that the difference in diet quality associated with statin use was evidenced among women aged ≥ 60 years and men ≥ 50 years raises concerns regarding residual risk of CVD, as this age group is the most at risk, even more so with MetS.^5^ Concerning the difference in the relationship between diet quality and statin use we observed between women and men, this translates to previously documented gender differences in health literacy and in perception of CVD risk as well as perception of the role of medication and diet in prevention of disease.^38^^,^^39^ Finally, the differential association between statin use and diet quality we observed according to annual household income has important implications for prevention of CVD among populations with lower socioeconomic status. Indeed, individuals with lower income are more likely to face economic barriers to healthy eating,^40^ while having greater low-cost access to preventive medication through Québec’s Public Prescription Drug Insurance.^41^ This duality between these 2 preventive modalities highlights how socioeconomic inequalities may influence both access and adherence to CVD preventive approaches. As such, further studies assessing how dynamics in the diet-statin relationship influences CVD incidence are needed. Complementing such analyses with economic assessments comparing the costs of implementing nutrition assistance programs aiming at improving diet quality with those associated with public preventive medication coverage among individuals with low incomes would provide a comprehensive perspective on the socioeconomic implications of the lack of complementarity between dietary and pharmacologic approaches.^6^^,^^39^

### Study limitations and strengths

Our study has to be interpreted within the context of its limitations and strengths. First, the main limitation of this work is related to the 2- to 3-year gap between plasma sample collection and medication assessment (2009-2010) and the completion of the FFQ (2012). Indeed, we could not assess whether medication or diet changed during this period. Similarly, because of the cross-sectional design, we could not assess the temporal dynamics between diet quality and medication use. Still, considering the inverse association we observed between the AHEI and plasma TG concentrations at the sample level suggests that diet did not significantly changed during this 2- to 3-year gap. Finally, the fact that LDL-C levels were estimated using the Friedewald formula likely introduced confounding into these data, considering that our sample was composed of individuals with relatively high TG levels. Limitations of the Friedewald calculation associated with high TG levels may be in cause in the unexpected positive relationship we observed between the AHEI and LDL-C levels.^42^ This is further supported by the lack of evidence of a relationship between the AHEI and non–HDL-C concentrations and by the negative relationship between the AHEI and TG concentrations that we observed at the sample level. Also, several clinical trials demonstrated that improving diet quality has cholesterol-lowering effects that are comparable with statins.^43^

With regard to strengths, the large sample size allowed to conduct subgroup analyses that revealed gender-, age-, socioeconomic- and health-associated factors modulating the diet-statin relationship. These findings are relevant for further development of targeted clinical and public health interventions promoting complementarity between diet and use of medication in prevention of CVD.

## Conclusions

In this cross-sectional study, adults with MetS using statins had slightly lower diet quality compared with those not using such medication. This difference was mostly caused by a lower consumption of vegetables and whole grains. Also, the lower diet quality among statin users was related to gender-, age-, socioeconomic-, and personal health-associated considerations. Our study sheds light on suboptimal complementarity between diet quality and use of cholesterol-lowering medication in the primary prevention of CVD in Québec.
