# Supplementary material for: Relationship Between Diet Quality and Statin Use Among Adults With Metabolic Syndrome From the CARTaGENE Cohort
Source: CJC Open. 2023 Sep 29;6(1):11–9. doi: 10.1016/j.cjco.2023.09.014 (PMC10837700; doi:10.1016/j.cjco.2023.09.014)
Supplement: Supplemental Figure and Tables [file mmc1.pdf]

Supplemental material

Relationship Between Diet Quality and Statin Use Among Adults With Metabolic Syndrome From the CARTaGENE Cohort

Amélie Bélanger, Clémence Desjardins, Lise Leblay, Mathieu Filiatrault, Olivier Barbier, Anne Gangloff, Jacinthe Leclerc, Jean Lefebvre, Arsène Zongo, Jean-Philippe Drouin-Chartier

Corresponding author:

Jean-Philippe Drouin-Chartier

jean-philippe.drouin-chartier@pha.ulaval.ca

Supplemental Table S1: Variables for which missing values were imputed.

**Variables**	**Imputed value**	**Imputations, n (%)**
Annual household income, $	50,000$ to 74,999$	123 (5.0)
Body mass index, kg/m^2	28.5	8 (0.3)
Education level	Technical school	13 (0.5)
HbA1c, %	5.60	18 (0.7)
IPAQ Physical activity level	Moderate	112 (4.5)
Plasma glucose, mmol/L	5.70	61 (2.5)
Self-reported history of high blood cholesterol	No	44 (1.8)
Self-reported history of high blood pressure	No	19 (0.8)
Smoking status	Never smoker	11 (0.4)
Waist circumference, cm	98.0	30 (1.2)

Supplemental Table S2: Alternate healthy eating index according to statin use and Canadian Cardiovascular Society criteria for statin indication.

Diet quality	**Participants not using statin and not meeting CCS statin indication criteria** (n=501)	**Participants not using statin and meeting CCS statin indication criteria** (n=1,517)	**Participants using statin** (n=463)	***P* value (between groups)**
AHEI total score (%)	41.4 (40.2, 42.6)	41.1 (40.3, 42.0)	40.0 (38.9, 41.2)	0.09

Statin indication was defined as: 1- Framingham risk score ≥20%; or 2- Framingham risk score between 10 and 19% with a) LDL-C≥3.50 mmol/L, or b) non-HDL-C≥4.3 mmol/L, or c) age≥50 years for men and ≥60 years for women. Criteria 2c needs concomitant presence of low HDL-C, high plasma glucose, high waist circumference, active smoking or hypertension. As all included participants had metabolic syndrome, these concomitant conditions are met;
Data are presented as adjusted mean (95% confidence interval). Multiple linear regression models were used (GLM procedure) with adjustments for gender, age, annual household income, smoking status, physical activity level, self-reported history of high blood pressure, body mass index, energy intake, and alcohol consumption;
Tukey-Kramer between groups *P* values:
Participants not using statin and not meeting CCS statin indication criteria vs participants not using statin and meeting CCS statin indication criteria, P=0.83;
Participants not using statin and not meeting CCS statin indication criteria vs participants using statin, *P*=0.13;
Participants not using statin and meeting CCS statin indication criteria vs participants using statin, *P*=0.11.

Supplemental Table S3: Relationship between the Alternative healthy eating index (AHEI) and concentrations of plasma lipids, according to statin use.

Plasma lipids	Participants not using statin (n=2,018)		Participants using statin (n=463)		*P* value (interaction)
	β (95% CI) per 10-point increment in the AHEI	*P* value	β (95% CI) per 10-point increment in the AHEI	*P* value	
Total-cholesterol (mmol/L)	0.04 (0.00, 0.08)	0.05	-0.03 (-0.12, 0.05)	0.43	0.12
LDL-cholesterol (mmol/L)	0.05 (0.01, 0.08)	0.01	0.02 (-0.05, 0.10)	0.53	0.58
HDL-cholesterol (mmol/L)	0.00 (-0.01, 0.01)	0.96	0.01 (-0.01, 0.04)	0.22	0.26
Non-HDL-cholesterol (mmol/L)	0.04 (0.00, 0.08)	0.04	-0.05 (-0.13, 0.03)	0.24	0.05
Triglycerides (mmol/L)	-0.01 (-0.05, 0.03)	0.51	-0.15 (-0.23, -0.07)	0.0001	0.001

Data are presented as mean (95% confidence interval). Linear regression models were used (GLM procedure) with adjustments for sex, age, annual household income, smoking status, physical activity level, self-reported history of high blood pressure, body mass index, energy intake, statin use, and alcohol consumption.

Supplemental Table S4: Odds ratios (95% confidence interval) of plasma cholesterol concentrations below the Canadian Cardiovascular Society targets for primary prevention of CVD associated with statin use and the Alternative healthy eating index (AHEI).

**Target**	**Exposure**	**Odds ratio (95% CI)**	***P* value**
LDL-C<2.00 mmol/L (n=205/2,481)	Statin use (yes vs no)	7.90 (5.68, 11.0)	<0.0001
	AHEI (per 10-points increment)	0.87 (0.75, 1.01)	0.07
Non-HDL-C<2.60 mmol/L (n=96/2,481)	Statin use (yes vs no)	6.04 (3.81, 9.57)	<0.0001
	AHEI (per 10-points increment)	0.98 (0.79, 1.21)	0.83

Data are presented as adjusted odds ratios (95% confidence interval). Logistic regression models were used (LOGISTIC procedure) with adjustments for sex, age, annual household income, smoking status, physical activity level, self-reported history of high blood pressure, body mass index, energy intake, and alcohol consumption.

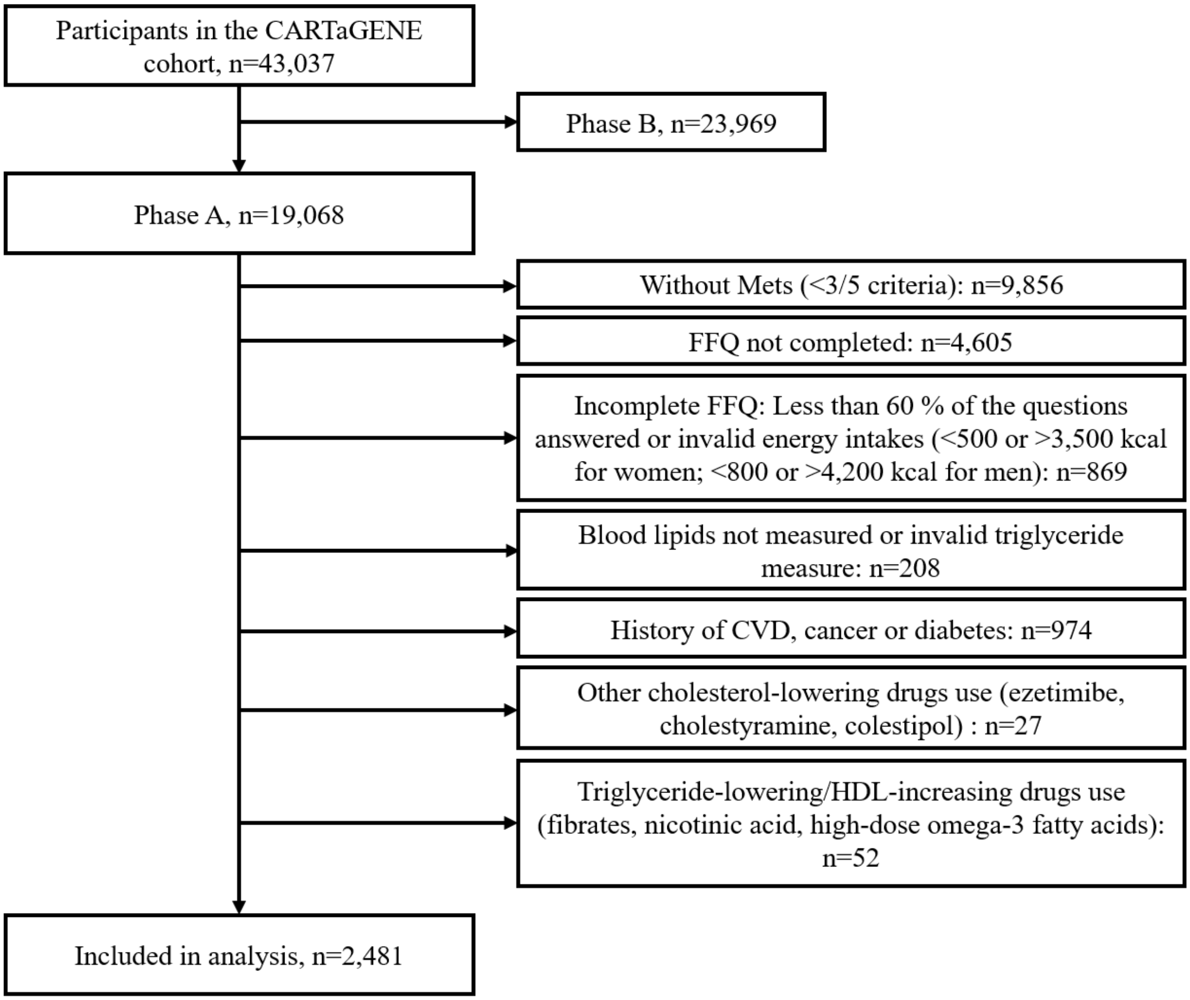

Supplemental Figure S1: Flow chart of participants selection.
